# Statins are Associated With a Reduced Risk of Brain Cancer: A Population-Based Case–Control Study

**DOI:** 10.1097/MD.0000000000003392

**Published:** 2016-04-29

**Authors:** Brian K. Chen, Hui-Fen Chiu, Chun-Yuh Yang

**Affiliations:** From the Department of Health Services Policy and Management, Arnold School of Public Health, University of South Carolina, Columbia, SC (BKC); Institute of Pharmacology, College of Medicine (H-FC); Department of Public Health, College of Health Sciences, Kaohsiung Medical University, Kaohsiung (C-YY); and Division of Environmental Health and Occupational Medicine, National Health Research Institute, Miaoli (C-YY), Taiwan.

## Abstract

The aim of this study was to investigate whether statin utilization is associated with brain cancer risk.

A population-based case–control study was conducted using nationally representative claims data from the National Health Insurance Bureau in Taiwan. Cases included all patients 50 years and older who received an index diagnosis of brain cancer between 2004 and 2011. Our controls were matched by age, sex, and index date. We estimated adjusted odds ratios (ORs) and 95% confidence intervals (CIs) using multiple logistic regression.

We examined 213 brain cancer cases and 852 controls. The unadjusted ORs for any statin prescription was 0.77 (95% CI = 0.50–1.18) and the adjusted OR was 0.59 (95% CI = 0.37–0.96). Compared with no use of statins, the adjusted ORs were 0.68 (95% CI = 0.38–1.24) for the group having been prescribed with statins with cumulative defined daily dose (DDD) below 144.67 DDDs and 0.50 (95% CI = 0.28–0.97) for the group with the cumulative statin use of 144.67 DDDs or more.

The results of this study suggest that statins may reduce the risk of brain cancer.

## INTRODUCTION

The antilipidemic properties of statins derive from their ability to inhibit 3-hydroxy-3-methyl glutaryl coenzyme A (HMG-CoA) reductase, a pivotal enzyme in the metabolic pathway of cholesterol synthesis.^1^ Statins are commonly prescribed to lower cholesterol levels, and numerous clinical trials have shown that they are effective in the primary and secondary prevention of myocardial infarction and cerebrovascular events.^2,3^ Their clinical effectiveness and the high prevalence of hyperlipidemia have led to widespread use of statins worldwide.

Meta-analyses of RCTs, however, found no evidence of an association between statin use and cancer incidence.^4,5^ Furthermore, rodent studies, however, suggest that statins are carcinogenic,^6^ with clinical trial evidence suggesting that statins could increase cancer risk in certain population.^7,8^ On the other hand, several studies of human cancer cell lines (including glioma cells) and animal tumor models show that statins may have chemopreventive properties by inhibiting cell cycle progression,^9^ inducing apotosis,^1,10^ suppressing angiogenesis,^11,12^ and arresting tumor growth and metastasis.^13–15^

Only 3 epidemiologic studies have examined the association between statin use and brain cancer risk. One cohort study did not find any association between more than 5 years of statin use and brain cancer risk.^16^ Two case–control studies reported that statin use was negatively associated with glioma risk.^17,18^ More recently, Gaist et al^19^ reported that statin use prior to diagnosis may reduce the death risk in glioblastoma patients. Statins are widely used worldwide, often on a chronic basis; however, available epidemiologic evidence evaluating the association between long-term statin use and brain cancer risk is limited. We examined the risk of brain cancer in relation to statin utilization based on a nationally representative administrative database from Taiwan.

## MATERIALS AND METHODS

### Data Source

On March 1, 1995, Taiwan implemented the National Health Insurance (NHI) program, a mandatory health insurance with universal access. The NHI covers 98% of the island's population receives for virtually all health care services, including outpatient and inpatient services, tradition Chinese medicine, dental care, childbirth, physical therapy, preventive health care, home care, and rehabilitation for chronic mental illness. Drawing from the wealth of healthcare utilization records covered under a single payer, the National Health Research Institute (NHRI) of Taiwan randomly sampled a nationally representative database of 1,000,000 individuals using a systematic sampling method for research purposes. According to the NHRI, no statistically significant differences between the sample and all enrollees with respect to age, gender, and healthcare costs.^20^ Comprehensive healthcare data include the enrollment files, the claim data, the Catastrophic Illness files, and the registry for drug prescription. Our dataset spans from January 1996 to December 2011, includes all claims generated by these 1,000,000 individuals, offering an excellent opportunity to explore the relationship between the use of statins and brain cancer risk. The database has been used successfully for numerous epidemiological studies, and data elements for prescription use, diagnoses, and hospitalizations have been demonstrated to be of high quality.^21,22^ This study was approved by the Ethics Review Board at the Kaohsiung Medical University Hospital (KMUH-IRB-exempt-20130032).

### Identification of Cases and Controls

Cases consisted of all individuals aged 50 years and older with an incident diagnosis of brain cancer (International Classification of Diseases, 9th revision, Clinical Modification [ICD-9-CM] Code 191) in an 8-year period between 2004 and 2011, who had no previous diagnosis of cancer Brain cancer diagnosis, were defined according to the Registry for Catastrophic Illness Patient Database, which is a separate subpart of the NHI database. The diagnosis of brain cancer in NHI database requires histologic confirmation to be reported in the Registry for Catastrophic Illness Patient Database.

We identified cases, who were newly diagnosed with brain cancer from January 1, 2004 with a margin of at least 8 years between January 1, 1996, to give sufficient time for case subjects to accumulate sufficient doses of statin to influence brain cancer development. The index date was defined as the date of first-time diagnosis of brain cancer. We excluded patients younger than age 50 because their low prevalence of statin use and low risk of developing brain cancer. Control subjects comprised hospital inpatients admitted to for diagnoses who were unrelated to statin use, such as orthopedic conditions, trauma (excluding wrist and hip fractures), and other conditions (acute infection, hernia, kidney stones, and cholecystitis).^23,24^ We excluded wrist and hip fractures because previous studies have reported a reduced risk of osteoporosis among statin users.^25–28^ We selected 4 control patients for every case patient. Control patients, who were without a previous cancer diagnosis, were matched to cases on sex, year of birth, and index date (case's brain cancer diagnosis date). To ensure that all cases and controls have similar opportunity before the index date, all patients included in this study were in the database since January 1, 1996. Our study prevented potential bias by choosing controls with exposure duration that were similar to the cases. In other words, cases and controls were matched on index date. For controls, the index date was defined as having a date of hospital admission in the same month of the index date of their matched case. Duration of follow-up was defined as the difference between the date of entry into the database and the index date. Assuming 80% power, significance level of 0.05, match of 4 controls to 1 case, and 20% of controls having received prescriptions for statins, 169 cases would be required to detect an odds ratio (OR) of 0.5.

### Exposure to Statins

All statin prescriptions were extracted from the NHRI medication prescription database. We collected information on the date of prescription, the daily dose, and the number of days supplied. We used the defined daily doses (DDDs) recommended by the WHO to quantify statin usages.^29^ Cumulative DDDs (cDDDs), which encompass both the dosage and duration of exposure, were estimated using the sum of dispensed DDD of any statins (lovastatin, pravastatin, rosuvastatin, fluvastatin, simvastatin, or atorvastatin) from January 1, 1996 to the index date. In our analysis, we categorized subjects into 1 of the 3 statins exposure types: nonusers (subjects without any statins prescription at any time between January 1, 1996 and the index date), moderate users (with doses equal to or below the median [144.67 cDDDs]), and heavy users (with doses above the median) based on the distribution of use among controls. We define patients who received a statin prescription between January 1, 1996 and June 30, 1996 (within 6 months of the patient's start in the data) as continuing statin users, whereas those who received a statin prescription after July 1, 1996 were defined as new statin users. We defined exposure to statins as patients who received at least 1 prescription for a statin at any time between January 1, 1996 and the index date. Cumulative DDDs is a time-dependent variable in which the supplies in days for each statin prescription dispensed were summed over the period from January 1, 1996 to the index date. We also examined the effect of the number of prescription fills. This variable was categorized as nonusers, number of prescription equal or below the median, and above the median based on the distribution of use among controls (0, 1–11, and >11).

### Potential Confounders

We obtained, for all subjects, information on potential confounders for the association between statin use and brain cancer, including comorbidities such as diabetes and stroke, recorded between January 1, 1996 and index date. Patients suffering from a stroke are frequently prescribed statins and undergo neuroimaging. The latter might in some instances coincidentally reveal brain cancer.^18^ We therefore regarded a history of stroke as a potential confounder. Diabetes is under intense scrutiny for its possible association with cancer.^18,30^ Diabetes is also associated with statin use and therefore was regarded as a potential confounder. Comorbid medical conditions were identified by the diagnosis codes that either occurred in the inpatient setting or appeared in at least 2 ambulatory care claims coded between January 1, 1996 and the index date. Radiation, head injury, seizures, and compromised immune system are possible risk factors for brain cancer. However, the attributing risk proportion of these risk factors is small and unlikely to be associated with statin use. We therefore choose not to adjust for these covariates. We also considered the effect of certain drugs to show positive results in chemoprevention including aspirin, nonsteroidal antiinflammatory drugs (NSAIDs), selective cyclooxygenase 2 (Cox2) inhibitors, and other lipid-lowering drugs (including fibrate, niacin, bile-acid binding resins, and miscellaneous).^31,32^ Patients with at least 1 prescription of the aforementioned drugs during the 1-year period prior to the index date as drug users of the respective medications. Furthermore, the number of hospitalizations 1 year before the index date entered or specification as a confounder.

### Statistical Analyses

We used Chi-square statistics to compare proportions between the cases and controls. To estimate the relative magnitude relative to the use of statins, we used a multiple conditional logistic regression model which was adjusted for number of hospitalization, continuing statin users, diabetes, stroke, use of aspirin, use of NSAID, use of Cox2, and use of other lipid-lowering drugs. We calculated ORs and their 95% confidence intervals (CIs) using nonusers as the reference. All analyses were performed using the SAS statistical package (version 9.2, SAS Institute Inc., Cary, NC). All statistical tests were 2-sided, with statistical significance at convential levels (*P* < 0.05).

### Sensitivity Analyses

To assess the impact of the latency period (the interval of time from tumor onset and clinical detection), we moved the index date to 1 year before brain cancer diagnosis or same date for matched control. We also redefined statin users as patients who received statins more than 28 cDDD between January 1, 1996 and the index date, whereas those who received fewer were defined as nonusers.^33^ The cut-point of 28 cDDD was chosen empirically as it corresponds to 28 days of usage (1 average statin prescription). These sensitivity analyses were conducted to evaluate the consistency of the results when applying different exposure definitions.

## RESULTS

We analyzed the association between statin use and brain cancer risk using records from 213 brain cancer cases and 852 selected matched controls. In Table 1, we present demographic characteristics and selected medical conditions of cases and controls. The mean age was 65.91 for cancer cases and 65.80 for the controls. The case group had a significantly higher rate of history of diabetes and stroke. However, the case group had a significantly lower rate of NSAIDs use. No significant difference was detected between cases and controls with respect to aspirin use, Cox2 use, and use of other cholesterol-lowering drugs.

**TABLE 1:**
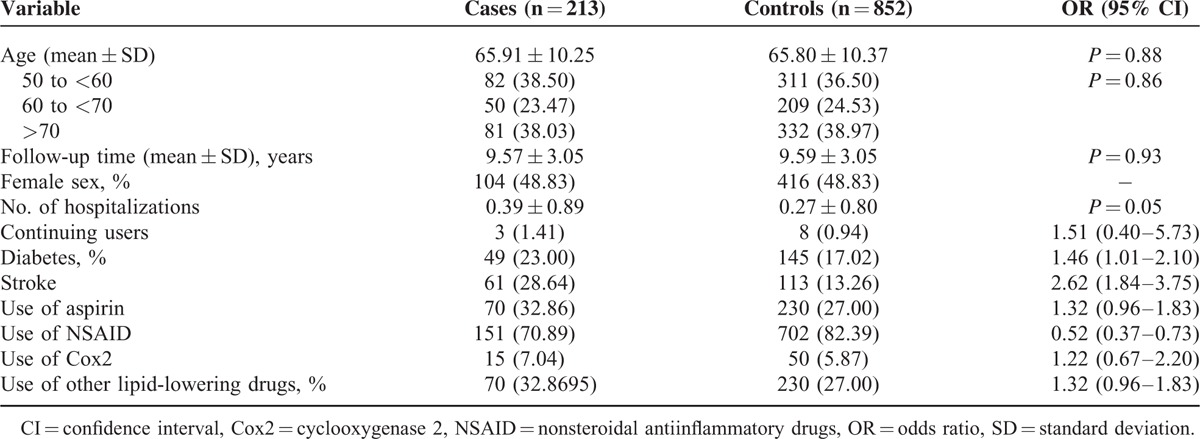
Demographic Characteristics of Brain Cancer Cases and Controls

The association between statin use and brain cancer is shown in Table 2. As noted in the table, 15.02% of the cases and 18.43% of the controls had used some quantity of at least 1 prescription for a statin. Having ever used any statins was associated with lower ORs for brain cancer risk (adjusted OR = 0.59, 95% CI = 0.37–0.96). When statin use was categorized by cDDD, the adjusted ORs were 0.68 (95% CI = 0.38–1.24) for the moderate users (with cDDDs below 144.67) and 0.50 (95% CI = 0.28–0.97) for heavy users (with cDDDs above 144.67) relative to nonusers. In addition, we detected a significant trend toward decreasing brain cancer risk with increasing cDDDs (X^2^ for linear trend = 5.01, *P* = 0.02). There was also a trend toward lower ORs (i.e., more risk reduction) with having more than 11 filled prescriptions than with 1 to 11 prescriptions as compared with nonusers.

**TABLE 2:**
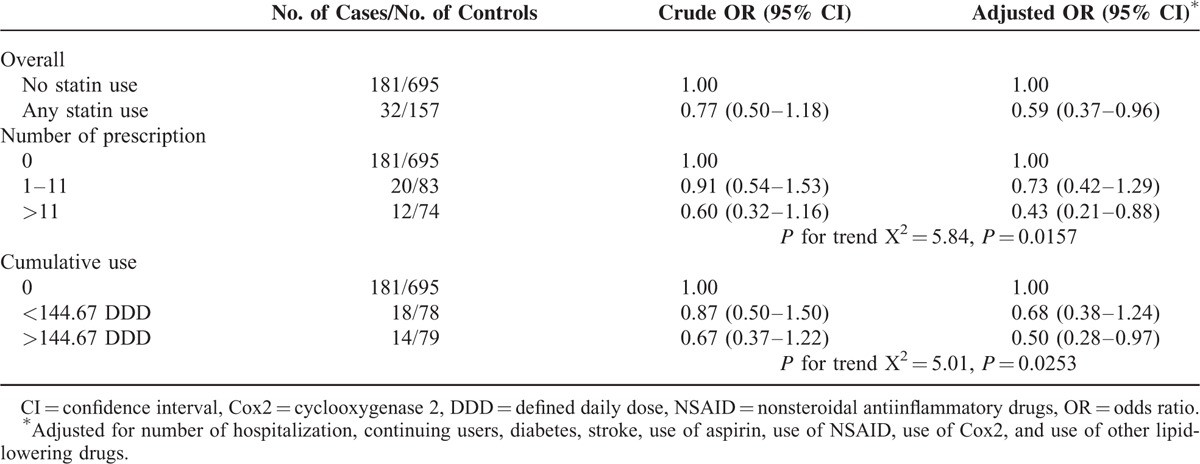
Associations Between Statin Use and Brain Cancer Risk in a Population-Based Case–Control Study, Taiwan, 2004 to 2011

A sensitivity analysis was conducted when we redefined exposure to statins as patients who received statins more than 28 cDDDs between January 1, 1996 and the index date, whereas those who received fewer were defined as nonusers. The results were similar. Statin users were associated with lower ORs for brain cancer risk (adjusted OR = 0.56, 95% CI = 0.33–0.96). When the analysis excluded statin prescriptions recorded within 1 year preceding the index date, statin users were associated with a decreased ORs for brain cancer risk (adjusted OR = 0.48, 95%CI = 0.28–0.80). The inverse associations were somewhat stronger.

## DISCUSSION

In our study based on nationally representative data from Taiwan, we found that the previous use of any statin was associated with a reduction in the risk of brain cancer. In addition, we documented a significant temporal trend toward greater cumulative statin dosages and lowered brain cancer risk, after controlling for observable confounders.

Recent observational studies link statins with beneficial effects in site-specific cancers, including breast,^34^ lung,^35^ and pancreatic cancers.^36^ However, none of these findings have been confirmed in large randomized controlled trials, and therefore these data cannot prove that statins prevent cancer.^5^ To the best of our knowledge, only 3 previous studies have investigated the association between statin use and brain cancer risk. In a population-based cohort study using the administrative health databases of Kaiser Permanente Medical Care Program (KPMCP) in northern California, Friedman et al^16^ found no relationship between over 5 years of statin use and brain cancer. This study was based on a very small sample (only 12 cases), and therefore had limited statistical power. In another case–control study in the United States with a larger sample size (458 cases vs 353 controls),^15^ the authors reported that statin use for >6 months was associated with a risk reduction in glioma (OR = 0.72, 95% CI = 0.52–1.00). As an interview study, recall bias was a potential drawback, with information collected from proxies in approximately 19% of cases. Recently, Gaist et al^18^ conducted a nationwide case–control study in Denmark and also reported an inverse relationship between long-term statin use and glioma risk (OR = 0.76, 95% CI = 0.59–0.98). Our findings are consistent with this study, arriving at similar conclusions (risk estimates for brain cancer) using similar methodology (a population-based case–control study design, and control for potential confounders such as use of aspirin, NSAIDs, and Cox2).

Our study results are consistent with the hypothesized biological mechanism of statins, although the exact causal pathway linking statin use with a reduction of brain cancer risk remains unclear. Prior studies have advanced several potential mechanisms, including: (1) Inhibition of derivative products of the mevalonate pathway, geranylgeranyl pyrophosphate (GGPP), and farnesylpyrophophosphate (FPP).^37,38^ Products of the mevalonate pathway GGPP and FPP play an important role in the activation of several cellular proteins, including small guanosine-5′-triphosphate binding proteins, such as K-ras, N-ras, and the Rho family.^37–40^ Statins are believed to disrupt the production of GGPP and FPP, thereby inhibiting the growth of cancerous cells, ultimately resulting in cell death.^1^ (2) Inhibition of the proteosome pathway activation, which reduces the breakdown of the cell cycle regulators p21 and p27. These molecules, in turn, exert their growth-inhibiting effects on cancer cell mitosis.^40–42^

For our analyses, we must not define a control disease that is associated with brain cancer and statin exposure. Doing so would force the cases and control groups to have similar proportion of statin use, an approach that could reduce the variations in the use of statins and limit our ability to identify the association between statin use and brain cancer risk. For this reason, we constructed a control group using diagnoses consisted not to be related to statin use. This approach, we believe, is necessary to increase methodological rigor and minimize selection bias. We defined exposure to statins as the cumulative sum of dispensed DDD of any statin from January 1, 1996 to the index date. In other words, we minimized potential selection bias by matching cases and controls on index date. To further increase our ability to match cases and controls, we chose controls from hospital inpatients, rather than a random sample from a panel of nationally representative 1,000,000 NHI enrollees without a cancer diagnosis. We chose not to conduct a nested case–control study given the low prevalence of statin use (19.2% in 2008, defined as the percentage of patients 50 years of age or older who had ever received at least 1 statin prescribe in the year), and the low incidence of brain cancer in the general population. However, in order to demonstrate a causal link, exposure to statins must precede the outcome of interest. From this perspective, the choice of our design (relative to a nested case–control design) should be considered a limitation.

A strength of our study is the use of an electronic database, which is drawn from the population and is highly representative. Statins were available with a prescription, so our data likely capture all statin use in Taiwan. Our study also benefits from the freedom from recall bias in the use of statins, because statin data were obtained from an historical database which collects all prescription information before the incidence of brain cancer. Furthermore, because of Taiwan comprehensive insurance coverage, and relatively small copayment (the copayment for prescription is about 10% of the cost of the drugs dispensed), Taiwanese patients have virtually no barriers to medical service due to accessibility and costs.^43^ As a result of this minimal barrier to medical access in Taiwan, we believe that the likelihood that the potential for nonprescription exposure (purchasing statins without a prescription) would be small.

We note several limitations of the present study. First, although we adjusted for some potential confounders in the statistical analysis, several confounding variables, including smoking,^44,45^ exposure to ionizing radiation, occupational exposure to solvents, and electromagnetic fields,^46^ were not included in our database. These variables are likely associated with brain cancer risk. However, we found no reason to believe that there is a correlation between the previously mentioned risk factors and the prescription of statins. Second, we were unable to contact the patients directly to enquire about their adherence to statin treatment because of encrypted identification number. The use of pharmacy dispensing data rather than actual usage data might have overestimated statin use. However, there is no reason to assume that this overestimation would differ systematically between cases and controls. In fact, if the patients did not adhere to their prescribed statin therapy, our findings would underestimate the effect of statin use. Third, several types of statins, including lovastain and pravastatin (available in 1990), simvastatin (available in 1992), and fluvastatin (available in April, 1996), were already available prior to patient enrollment in the database. Prescriptions for these drugs prior to 1996 would not be captured in our analysis, resulting in potential underestimation of the cumulative DDDs and bias the observed association downwards. However, only 1.41% of the cases (3 cases) and 0.94% of the controls (8 cases) were continuing statin users (defined as patients who received a statin prescription between January 1, 1996 and June 30, 1996, i.e., within 6 months of the patient's first usage of statin prescription in the data). Furthermore, since there was no significant difference between cases and controls, the potential underestimation of exposure should be small and should not be differential between cases and controls. In addition, some exposure misclassification could potentially result from the fact that information on prescription was available only after 1996. Fourth, we are unable to analyze the risks separately for users of distinct statins due to the relatively small number of cases and statin users. Fifth, identification of brain cancer or any other comorbid medical conditions as well as prescription information relying on administrative claims data may be less accurate than those obtained using standardized criteria. However, this misclassification is again likely to be nondifferential (i.e., there is no reason to assume that this would be different for cases and controls) and would therefore tend to underestimate rather than overestimate the true association. Sixth, this study was based on 32 statin exposed cases, and therefore limited statistical power (power = 65.32%) should be considered when interpreting the study results. Finally, as with any observational study, residual confounding by unmeasured factors which are different between cases and controls is also possible. However, we controlled for the confounding effect of medical attention by adding the number of hospitalizations in the conditional logistic regression model.

In summary, our study suggests that statin use may reduce the risk of brain cancer. Further studies, particularly prospective randomized trial studies, are necessary to confirm our findings and the value of statins in brain cancer prevention and treatment.
